# Draft Genome Sequence of *Endozoicomonas acroporae* Strain Acr-14^T^, Isolated from *Acropora* Coral

**DOI:** 10.1128/genomeA.01576-17

**Published:** 2018-02-08

**Authors:** Kshitij Tandon, Pei-Wen Chiang, Wen-Ming Chen, Sen-Lin Tang

**Affiliations:** a Biodiversity Research Center, Academia Sinica, Taipei, Taiwan; b Bioinformatics Program, Institute of Information Science, Taiwan International Graduate Program, Academia Sinica, Taipei, Taiwan; c Institute of Bioinformatics and Structural Biology, National Tsing Hua University, Hsinchu, Taiwan; d Laboratory of Microbiology, Department of Seafood Science, National Kaohsiung Marine University, Nantzu, Kaohsiung City, Taiwan

## Abstract

A lacuna exists in our understanding of the genetic makeup of *Endozoicomonas* bacteria, due to scarcity of genome sequences. We report here the first draft genome sequence of *Endozoicomonas acroporae* Acr-14, a type strain isolated from the coral *Acropora*. This sequence will foster an understanding of the genetic makeup and role of hosts in shaping gene repertoires.

## GENOME ANNOUNCEMENT

The genus *Endozoicomonas* belongs to the phylum *Proteobacteria*, class *Gammaproteobacteria*, with the type species being *Endozoicomonas elysicola* (1). Members of this genus form a significant proportion of the bacterial community in diverse marine hosts, including sponges (2), mollusks (1, 3–5) tubeworms (6), gorgonians (7, 8), and corals (9–14). Currently, there are only two whole-genome sequences of *Endozoicomonas montiporae* strains isolated from the Scleractinia coral *Montipora aequituberculata* (15, 16). Here, we report the first draft genome sequence of strain Acr-14^T^ from the novel species *Endozoicomonas acroporae*, isolated from *Acropora* coral off the coast of southern Taiwan. The type strain and taxonomic information have been reported previously (17).

Strain Acr-14^T^ was purified and cultivated as described previously (17), genomic DNA was isolated with the cetyltrimethylammonium bromide (CTAB) method, and the purity of DNA was checked with NanoDrop 1000 (Thermo Scientific, USA). Whole-genome sequencing was performed at the Biodiversity Research Center Academia Sinica (BRCAS) core sequencing facility with a paired-end MiSeq library generated to achieve a 500-bp insert size with an Illumina MiSeq platform. The obtained reads were quality filtered and trimmed (at a Phred score of ≥30) with the NGS QC toolkit (18). Quality-filtered and trimmed reads were *de novo* assembled using CLC Genomics Workbench version 1.10.1 (Qiagen), with a bubble size of 40, automatic detection of word size enabled, and a minimum contig length of 500 bp (no scaffolding was performed). A total of 309 contigs yielded a genome sequence 6,048,850 bp long, with 448× coverage and a G+C content of 49.16%. The largest contig and *N*_50_ value were 161,511 bp and 47,658 bp, respectively. The genome was estimated to be 98.56% complete using CheckM (19).

Open reading frame (ORF) prediction and automatic annotation were performed using the Prokka software (20), with default parameters, obtaining the outputs in GenBank format. The genome contains 5,104 genes, 5,018 coding sequences (CDSs), 79 tRNAs, 5 rRNAs (16S and 5S), and 4 repeat regions.

Furthermore, 5 prophages (2 intact, 2 incomplete, and 1 questionable) were detected in the genome using PHAST (21), and 4 typical clustered regularly interspaced short palindromic repeat (CRISPR) structures were also detected with Prokka (20).

A BLASTn (22) comparison of 16S rRNA obtained after assembly with the NR database gave an identical (100%) match with the already-deposited type strain 16S rRNA (GenBank accession no. LN875493). According to phylogenetic analysis based on 16S rRNA, the closest relative of Acr-14^T^ is *Endozoicomonas atrinae* WP70^T^ (sequence similarity, 96.7%) (17).

### Accession number(s).

This whole-genome shotgun project has been deposited at DDBJ/ENA/GenBank under the accession no. PJPV00000000. The version described in this paper is version PJPV01000000.
